# Ginsenoside Rd Ameliorates Auditory Cortex Injury Associated With Military Aviation Noise-Induced Hearing Loss by Activating SIRT1/PGC-1α Signaling Pathway

**DOI:** 10.3389/fphys.2020.00788

**Published:** 2020-07-21

**Authors:** Xue-min Chen, Shuai-fei Ji, Yu-hui Liu, Xin-miao Xue, Jin Xu, Zheng-hui Gu, Sen-lin Deng, Cheng-dong Liu, Han Wang, Yao-ming Chang, Xiao-cheng Wang

**Affiliations:** ^1^Department of Aerospace Hygiene, School of Aerospace Medicine, Air Force Medical University, Xi’an, China; ^2^Medical School of Chinese PLA, Research Center for Tissue Repair and Regeneration Affiliated to the Medical Innovation Research Department and 4th Medical Center, PLA General Hospital, Beijing, China; ^3^Center of Clinical Aerospace Medicine, School of Aerospace Medicine, Key Laboratory of Aerospace Medicine of Ministry of Education, Air Force Medical University, Xi’an, China; ^4^School of Basic Medicine, Air Force Medical University, Xi’an, China; ^5^Department of Cardiothoracic Surgery, Changzheng Hospital, Naval Medical University, Shanghai, China

**Keywords:** ginsenoside Rd, noise-induced hearing loss, auditory cortex, sirtuin 1, proliferator-activated receptor-gamma coactivator 1α

## Abstract

Free radicals and oxidative stress play an important role in the pathogenesis of noise-induced hearing loss (NIHL). Some ginseng monomers showed certain therapeutic effects in NIHL by scavenging free radicals. Therefore, we hypothesized that ginsenoside Rd (GSRd) may exert neuroprotective effects after noise-induced auditory system damage through a mechanism involving the SIRT1/PGC-1α signaling pathway. Forty-eight guinea pigs were randomly divided into four equal groups (normal control group, noise group, experimental group that received GSRd dissolved in glycerin through an intraperitoneal injection at a dose of 30 mg/kg body weight from 5 days before noise exposure until the end of the noise exposure period, and experimental control group). Hearing levels were examined by auditory brainstem response (ABR) and distortion product otoacoustic emission (DPOAE). Hematoxylin–eosin and Nissl staining were used to examine neuron morphology. RT-qPCR and western blotting analysis were used to examine SIRT1/PGC-1α signaling and apoptosis-related genes, including Bax and Bcl-2, in the auditory cortex. Bax and Bcl-2 expression was assessed via immunohistochemistry analysis. Superoxide dismutase (SOD), malondialdehyde (MDA), and glutathione peroxidase (GSH-Px) levels were determined using a commercial testing kit. Noise exposure was found to up-regulate ABR threshold and down-regulate DPOAE amplitudes, with prominent morphologic changes and apoptosis of the auditory cortex neurons (*p* < 0.01). GSRd treatment restored hearing loss and remarkably alleviated morphological changes or apoptosis (*p* < 0.01), concomitantly increasing Bcl-2 expression and decreasing Bax expression (*p* < 0.05). Moreover, GSRd increased SOD and GSH-Px levels and decreased MDA levels, which alleviated oxidative stress damage and activated SIRT1/PGC-1α signaling pathway. Taken together, our findings suggest that GSRd ameliorates auditory cortex injury associated with military aviation NIHL by activating the SIRT1/PGC-1α signaling pathway, which can be an attractive pharmacological target for the development of novel drugs for NIHL treatment.

## Introduction

Noise, such as traffic noise, industrial noise, construction noise, and living noise, is a factor that seriously endangers people’s health, especially through serious auditory system damage that may result in NIHL. Soldiers are often exposed to high-intensity noise generated by weapons and equipment in military operations and daily training (Rajguru, 2013); therefore, they have a high incidence of noise damage, especially those involved in aviation units. Currently, the most common preventive measure against NIHL is wearing hearing protection devices. However, the aviation unit ground crew is often unable or unwilling to wear these devices, including earplugs, due to poor compliance and comfort. Therefore, their auditory systems are vulnerable to noise damage and they have a high prevalence of hearing system diseases, such as NIHL, tinnitus, and auditory allergy (Miao et al., 2019). Therefore, research on supplements for hearing protection devices that would provide a comprehensive protection and reduce the incidence of NIHL and other diseases in personnel working in a noisy environment, especially helicopter units and ground service personnel, is mandatory.

Metabolic theory is recognized as one of the three main mechanisms of noise-induced damage to the auditory system. Noise exposure leads to excessive free radicals, lipid peroxidation, glutamic acid excitatory toxicity, and calcium overload, resulting in mitochondrial damage; protein, lipid, and DNA damage; serious disorders of hair cells and Sertoli cell enzymes; oxygen and energy metabolism disorders; cell necrosis and apoptosis; all these factors ultimately lead to degeneration, death, and loss of inner ear cells and neurons (Zhang et al., 2017). Several studies have confirmed that noise-induced excessive free radicals in the cochlea and auditory cortex play an important role in the occurrence and development of NIHL (Tuerdi et al., 2017). Besides, sirtuin 1 (SIRT1) is involved in the regulation of autophagy by increasing mitochondrial activity and reducing oxidative stress. Moreover, it deacetylates proliferator-activated receptor-gamma coactivator 1α (PGC-1α), which may be associated with noise-related ROS generation. Therefore, pharmaceutical studies based on the above mechanisms are currently being conducted to develop supplements for hearing protection devices.

Since antioxidants or substrates could prevent and treat NIHL by scavenging free radicals, maintaining ROS balance, and limiting lipid peroxidation, increasing and supplementing endogenous and exogenous antioxidants, such as glutathione (GSH), acetylcysteine (NAC), vitamin A, C, and E, and hydrogen-rich water, could reverse the structural and functional impairment of the auditory system caused by noise of various spectrum and intensity (Bielefeld et al., 2007; Angrini and Leslie, 2012; Zhou et al., 2012; Choi et al., 2014; Kurioka et al., 2014). Moreover, some traditional Chinese medicine components, such as ginsenoside, act as antioxidants that scavenge free radicals. Ginsenoside is the most important active ingredient in Panax ginseng and Panax notoginseng, and isolation of its effective monomers is helpful toward the elucidation of its pharmacological effects. GSRd is a saponin monomer with a relatively lower content, but better pharmacological activity, that has been reported to scavenge free radicals and exert antioxidant, anti-aging, and analgesic effects (Leung and Wong, 2010).

However, there have been no mechanistic studies on the protective effect of GSRd against NIHL, especially against the damage caused by high-intensity and specific frequency noise in military aviation to the auditory system. We hypothesized that the SIRT1/PGC-1α signaling pathway may be involved in the protective effect of GSRd against NIHL. Therefore, we aimed to establish animal models of NIHL by playing high-intensity specific spectrum noise collected from a military helicopter in China. These animal models were used to assess the protective effect of GSRd in noise-induced structural and functional damage to the auditory cortex, clarify the involvement of the SIRT1/PGC-1α signaling pathway, and suggest new ideas for NIHL prevention and treatment.

## Materials and Methods

### Animal Groups

Forty-eight pigmented male guinea pigs weighing 250–300 g were purchased from the Laboratory Animal Center of Air Force Medical University (AFMU) of China. All animals were confirmed to have intact Preyer’s reflex, normal tympanic membrane, and were free from otitis media. They were kept in a temperature-controlled room at 20–25°C with a 12 h light/dark cycle, 60–65% air relative humidity, and free access to food and water. Before the experiments began, they were allowed 5 days to adapt to the new living conditions. GSRd (purity 98%; Tai-He Biopharmaceutical Co. Ltd, Guangzhou, China) was kindly provided by Department of Neurology, Xijing Hospital in Xi’an, China.

Guinea pigs were randomly divided into four groups of 12 animals each. Group 1: normal control group (Con), included animals that did not receive any noise and treatments. Group 2: noise group (NE), included animals that were exposed to military aviation noise, but received no treatment. Group 3: experimental group (Rd), included animals that were exposed to military aviation noise and received GSRd dissolved in glycerin through an intraperitoneal (i.p) injection at a dose of 30 mg/kg body weight (Shi et al., 2008; Zhang et al., 2014). GSRd was administered for 5 days before noise exposure and for 10 days until the end of the noise exposure period (Henderson et al., 2006; Lu et al., 2014). Group 4: experimental control group (Vehl), included animals that were exposed to military aviation noise and received 30 mg/kg glycerin i.p injections at similar timings as the Rd group. All procedures were approved by the Institutional Animal Care and Use Committee of AFMU in Xi’an, China.

### Noise Exposure and Procedures

In the experiment, we collected broad band noise of the engine starting of a certain type of helicopter in China’s aviation army. Noise was played continuously through a loudspeaker (Soundtop SF-12, Jia-sheng Audio Equipment Co. Ltd, Guangzhou, China) and amplified by a low-distortion power amplifier (Soundtop QA-700, Jia-sheng Audio Equipment Co. Ltd, Guangzhou, China). The experiment was implemented in a soundproof room with an air conditioner to moisten the air and increase local ventilation. The animals were placed unrestrained in a cage 30 cm away from the loud speaker (Heng-sheng Electronics, Jiaxing, China) through a condenser microphone every time before experiment to ensure that they received noise exposure of equal intensity and the sound intensity was less than 3 dB within the animals’ range of motion. The animals of NE, Rd, and Vehl groups were exposed to the noise at a level of 115 dB from 10 a.m. to 14 p.m. for 5 days (Chen et al., 2019). The control group did not receive noise stimulation, and the original cage background noise was <20 dB, while other conditions were similar to those of the experimental groups.

### Auditory Brainstem Response (ABR) Measurements

Guinea pigs were subjected to ABR measurements (Otometrics, Taastrup, Denmark) before, 1 h, 1 day, 3 days, and 5 days after the noise stimulation. Subsequently, they were anesthetized by i.p injection of 4% pentobarbital (0.3 mL/100 g). After anesthesia, they were moved into a soundproof room and placed on an electric blanket. Cotton swabs were used to clear cerumen from both ears. The recording electrode was inserted subcutaneously into the middle of the vertical line between the ears on the head, the reference electrode was inserted subcutaneously behind the test ear, and the grounding electrode was inserted into the root of the right hind limb. The sound stimulus was composed of a 15-ms tone burst, with a rise-fall time of 1 ms at frequencies of 2, 4, and 8 kHz (Su et al., 2019). Brainstem auditory evoked responses, which were accumulated 600 times, were stimulated by density alternating short click sounds produced by the potentiometer. The signal was monitored by an oscilloscope and then input into a computer through an amplifier. The lowest stimulation intensity that allowed the ABR III wave to be distinguished was considered as the auditory threshold.

### Distortion Product Otoacoustic Emission (DPOAE) Measurements

After each ABR test, the Smart OAE system (Intelligent Hearing Systems, Miami, FL United States) was used for DPOAE test. Anesthetized guinea pigs were placed in a soundproof room. An appropriate size of the earpiece was selected and gently inserted into the external auditory canal of the guinea pigs to the best possible fit. A high-frequency transducer was used to deliver the primary tones to the ear canal through a flexible probe. The stimulus intensity at *f*1 was 65 dB SPL and at *f*2 was 60 dB SPL, with the *f*2/*f*1 ratio set at 1.22. Next, we selected the 2*f*1 – *f*2 DPOAE amplitudes for testing. A total of eight test points within the 0.5–8 kHz range were selected (Table 1) (Chen et al., 2019). The absolute DPOAE signal amplitudes were automatically recorded simultaneously with the noise amplitudes using the instrument. The relative amplitude of DPOAE, namely the signal-to-noise ratio, was obtained by subtracting the absolute signal amplitudes from the noise amplitudes, and values > 3 dB were considered significant.

**TABLE 1:** Results at eight frequency points with DPOAE (Hz).

Frequency	Frequency points							
	1	2	3	4	5	6	7	8
*f*1	453	679	905	1811	2716	3621	5432	7242
*f*2	552	828	1104	2209	3314	4418	6627	8837
2*f*1–*f*2	354	530	706	1413	2118	2824	4237	5647
$\sqrt{f1f2}$	500	750	1000	2000	3000	4000	6000	8000

### Tissue Preparation

After determination of ABR and DPOAE, the chest of each guinea pig was quickly opened. From the apex of the heart, a perfused pillow was inserted, 0.9% saline injected, and the right auricle was scissored. After full perfusion, the guinea pig was decapitated, and the brain was taken out and placed on ice. Anatomical localization of the auditory cortex was performed as previously described (Wallace et al., 2000; Schofield and Coomes, 2005). In each group, three brains were postfixed in 4% (w/v) paraformaldehyde solution for 5 days to allow paraffin sectioning. The left and right brain hemispheres were separately embedded in paraffin and 5 μm longitudinal coronal sections were prepared for HE staining, Nissl staining, and immunohistochemical investigation. The left and right auditory cortices of the other six brains were dissected and preserved at −80°C, with the left auditory cortex being used for western blotting and the right auditory cortex being used for RT-qPCR analysis. The left and right brain hemispheres were preserved, respectively, for detection of SOD activity, MDA level, and GSH-Px level.

### Hematoxylin–Eosin Staining

Hematoxylin–eosin staining was conducted as described by Su et al. (2019). After deparaffinization in xylene and rehydration in gradient alcohol, 5 μm longitudinal coronal sections were stained with hematoxylin solution for 2 min and then rinsed in distilled water for 1 min. After differentiation with 1% acid-ethanol (1% HCl in 70% ethanol), sections were stained with eosin solution for 20 s, re-immersed in graded alcohol, and cleared in xylene. Slides were mounted and photographed using a light microscope (Olympus, Tokyo, Japan).

### Nissl Staining

Nissl staining was carried out according to the method proposed by Su et al. (2019). Sections were deparaffinized using xylene, hydrated with 95%, 85%, and 70% gradient alcohol, and rinsed using running tap water for 2 min. Subsequently, they were stained using 1% toluidine blue dye at room temperature for 10 min and then rinsed in distilled water for 1 min, followed by dehydration with gradient alcohol, clearing in xylene, and sealing with neutral gum. Nissl-positive cells were then viewed under the light microscope. Five representative high power visual fields were collected from each section, and positive cells were counted under the light microscope (×200).

### Immunohistochemical Investigation

Immunohistochemical investigation was carried out as described by Cheng et al. (2017) using the DAB staining kit. Brain sections were deparaffinized and hydrated, and then rinsed in distilled water (twice for 5 min each). Next, sections were treated with Citrate-EDTA Antigen Retrieval Solution (Beyotime Biotechnology Co. Ltd, Shanghai, China) in a water bath for 20 min at 95–100°C. After cooling to room temperature, sections were rinsed in PBS (three times for 5 min each). Immunohistochemistry analyses were then performed following the manufacturer instructions for SPlink Detection Kits, namely Biotin-Streptavidin Horseradish Peroxidase (HRP) Detection Systems (Zhongshan Golden Bridge Biotechnology Co. Ltd, Beijing, China). A proper amount of endogenous peroxidase blocker was added, and the sections were incubated at room temperature for 10 min followed by rinsing in PBS (three times for 5 min each). Then, 100 μL of goat serum albumin was added, sections were incubated at room temperature for 10–15 min, and the serum was carefully poured out not to wash the sections. Sections were then incubated in a thermostat with rabbit polyclonal antibodies against B-cell lymphoma-2 (Bcl-2, 1:200, Wanleibio, China) and Bcl-2 associated X protein (Bax, 1:200, Wanleibio, China) at 37°C for 1 h. Biotin-labeled goat anti-rabbit IgG polymer and HRP labeled Streptomyces ovalbumin working fluid were then sequentially added to the sections for 15 min each. DAB was then used for color development. Finally, the sections were dehydrated using gradient alcohol, cleared in xylene, and cover slipped with neutral gum for light microscopic observation. The optical density and the total area of DAB-positive staining were analyzed by ImageJ 1.51 (Wayne Rasband, National Institutes of Health, United States).

### Pharmacological Effect of GSRd and Functional Enrichment Analyses of Differentially Expressed Genes After SIRT1 Activation via Bioinformatic Analysis

We predicted GSRd pharmacological effects and the pathways associated with SIRT1 activation by bioinformatic analysis. STITCH database^1^ (Szklarczyk et al., 2016) was mined to explore the pharmacological effects of GSRd and further conduct functional enrichment analyses. The gene expression profiles after SIRT1 activation in nervous cells were collected from the Gene Expression Omnibus (GEO) (data accessed at GEO: GSE39551). An absolute log2-fold change (|FC|) > 1 and a *p* value < 0.05 were set as cut-off criteria of differentially expressed genes (DEGs). Then, functional enrichment analyses of DEGs were conducted via metascape database^2^ (Zhou et al., 2019).

### Quantitative Real-Time PCR (RT-qPCR) Analysis

RT-qPCR analysis experimental steps proposed by Zhang et al. (2020) were used for reference. To investigate whether the protective effects of GSRd on NIHL were mediated via an effect on the apoptotic and SIRT1/PGC-1α pathway, RT-qPCR assays was performed to examine the changes in mRNA levels of different molecules in the auditory cortex of guinea pigs. The auditory cortices were pulverized and total RNA was extracted using RNAiso (TaKaRa, Tokyo, Japan). The purity and concentration of total RNA were determined using an ultraviolet spectrophotometer, and samples showing an OD260 to OD280 ratio between 1.8 and 2.0 were used for reverse transcription. The extracted total RNA was diluted to 300–500 ng/μL, the amount of total RNA was determined by its concentration, and then combined with 2 μL 5 × Primer Script RT Master Mix (TaKaRa, Tokyo, Japan). RNase-free bidistilled water was added up to 10 μL and the mixture at 37°C for 15 min and then at 85°C for 5 s to synthesize the complementary DNA (cDNA) template. A total of 2 μL of the cDNA template was mixed with 12.5 μL of SYBR Premix Ex Taq II (2×) (TaKaRa, Tokyo, Japan), 8.5 μL of sterile distilled water, and 1 μL of the forward and reverse primers, in that respective order (Table 2, Sangon Biotechnology Co. Ltd, Shanghai, China). The target gene was then reverse transcribed and amplified under the following conditions: 95°C for 30 s, 40 cycles of 95°C for 5 s and 60°C for 30 s, and a final step of 95°C for 15 s. GAPDH was used as an internal control and the relative expression of the target gene was calculated using the 2^–ΔΔ*Ct*^ method.

**TABLE 2:** Sequences of primers used in this study.

Gene name	Forward (5′–3′)	Reverse (5′–3′)
SIRT1	CGTTGGAACAAGTTGCAGGAATCC	TCCTCGTACAGCTTCACAGTCAAC
PGC-1α	GACACAACACGGACAGAACTGAGG	GCATCACAGGTGTAACGGTAGGTG
Bax	TCGCTGATGGCAACTTCAACTGG	GGCGGTCTCGGAGGAAGTCTAG
Bcl-2	CCAAGACTTCGCTGAGATGTCCAG	GGCGATGTTGTCCACCAGAGG
GAPDH	GGAAGCTGTGGCGTGATGGC	TTCTCCAGGCGGCAGGTCAG

### Western Blotting Analysis

The experimental steps of western blotting analysis refer to the articles previously published by our teaching and research department (Cheng et al., 2017; Su et al., 2019). Total auditory cortex protein was extracted with 500 μL protein extraction reagent (78505, Thermo Scientific, Rockford, IL, United States) containing 5 mM PMSF, centrifuged at 12,000 rpm for 10 min at 4°C, and the supernatant collected. The total protein concentration was determined using the BCA Protein Assay Kit (23250, Thermo Scientific) in accordance with the manufacturer’s instructions. Then, 30 μg of protein from each group was loaded to 4–12% Bis-Tris PAGE gels under denaturing conditions in the NuPAGE Bis-Tris Pre-cast Gel System (Invitrogen Life Technologies, Carlsbad, CA, United States). After electrophoresis, proteins were transferred to a polyvinylidene fluoride membrane (0.45 μm, Millipore, Darmstadt, Germany) in an XCell Blot Module transfer system (Invitrogen Life Technologies). Membranes were blocked in 5% fat-free milk powder in PBS containing 0.1% (w/v) Tween 20 (PBST) for 2 h at room temperature, and then incubated overnight at 4°C with rabbit polyclonal antibody against SIRT1 (1:1000, Wanleibio, China), PGC-1α (1:1000, Wanleibio), Bax (1:1000, Wanleibio), or Bcl-2 (1:1000, Wanleibio, China), with β-actin (1:1000, Wanleibio) as an internal control. The membranes were rinsed with PBST six times for 5 min each and incubated in PBST with HRP conjugated goat anti-rabbit IgG (1:10,000, Zhuangzhi Biotechnology Co. Ltd, Xi’an, China) for 1 h at room temperature, and then rinsed again as described. Proteins on the membranes were detected by enhanced chemiluminescence detection reagents (Millipore, Billerica, MA, United States) in a Gel Image Analyzing System (Tanon Science & Technology, Shanghai, China). Finally, the expression of each protein was measured using ImageJ 1.51.

### Detection of SOD Activity, MDA Level, and GSH-Px Level

Superoxide dismutase activity represented the ability to scavenge free radicals as that of antioxidant enzymes. MDA represented the level of free radicals based on lipid peroxidation. GSH-Px represented the ability of selenium to remove harmful peroxide metabolites. The guinea pig auditory cortex samples were prepared as a 10% homogenate with 0.9% normal saline and centrifuged at 3000 rpm at 4°C for 10 min, and the supernatants were subsequently collected. All three samples were examined using the commercial testing kit (Jiancheng Biotechnology Co. Ltd, Nanjing, China) according to manufacturer instructions. The chromaticity of each group was monitored by a microplate reader (Thermo Fisher, Waltham, MA, United States) at 450 nm for SOD activity, 532 nm for MDA level, and 412 nm for GSH-Px level. One unit of SOD was defined as the amount of enzyme needed to inhibit superoxide production by 50%.

### Statistical Analysis

SPSS 23.0 (IBM Corp., Armonk, NY, United States) and GraphPad Prism 7.0 (GraphPad Software, Inc., San Diego, CA, United States) packages were used to analyze data and make charts, respectively. The hearing level, section staining results, SOD and MDA activity, and mRNA and protein expression were analyzed using one-way ANOVA. Dunnett’s *t*-test was used for comparisons between the experimental groups and the control group. Statistical significance was set at *p* < 0.05 and the results are presented as means ± SE.

## Results

### Bioinformatics Analysis for Pharmacological Effect of GSRd and Gene Expression Profiles After SIRT1 Activation in Nervous Cells

The information on GSRd from the STITCH database showed that the targeted GSRd genes are mainly associated with apoptosis regulation, mitochondrial activity, and oxidative stress response (Supplementary Figure S1). Biological process (GO) uncovered that GSRd has the ability to regulate positively the apoptosis process by various means (Supplementary Figure S2); furthermore, KEGG pathway suggested that targeted GSRd genes are close linked to apoptosis-associated pathways (Supplementary Figure S3). Microarray dataset GSE39551 was obtained from the GEO database to explore gene expression profiles after SIRT1 activation. A total of four samples were analyzed, including two with resveratrol (SIRT1 activator) treatment and two with DMSO treatment. Finally, there were 352 DEGs, including 216 up-regulated genes and 136 down-regulated genes (Supplementary Figure S4). According to the functional enrichment analysis of DEGs (Supplementary Table S1 and Supplementary Figure S4), DEGs caused by SIRT1 activation mainly positively regulate the metabolic process of ROS, and that regulation comes in first. The gene count about positive regulation of programmed cell death comes in first, up to 19. Therefore, bioinformatics analysis showed that both GSRd and SIRT1 activation exhibited the alleviation of oxidative stress and anti-apoptosis effect; therefore, there may be an underlying biological link between them.

### ABR Threshold Values

**FIGURE 1:**
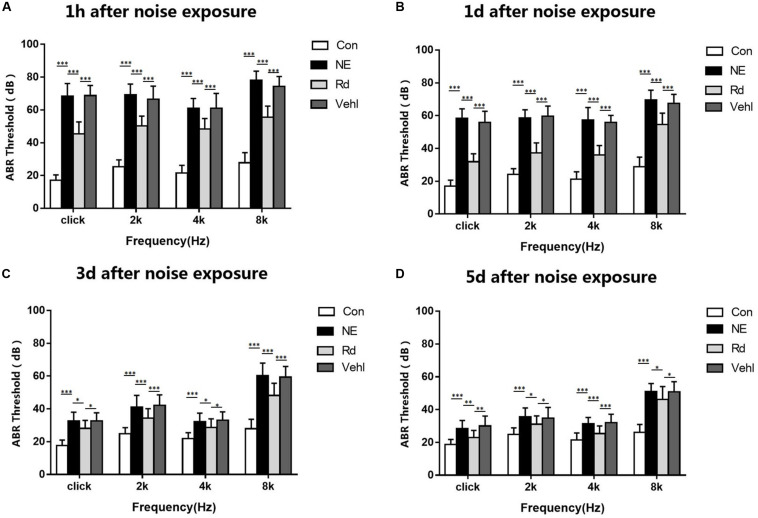
The average ABR threshold values in all groups 1h **(A)**, 1d **(B)**, 3d **(C)** and 5d **(D)** after 115 dB(A) noise exposure. *p < 0.05, **p < 0.01, ***p < 0.001.

The ABR threshold values are shown in Figure 1. Prior to noise stimulation, there was no significant difference in the ABR threshold values among the groups. Compared with those in the Con group, the average ABR threshold values of the NE, Rd, and Vehl groups were increased significantly at all frequencies examined at a noise exposure of 115 dB (A) (*p* < 0.01). The threshold value across the tested stimuli ranged from 30 to 50 dB. Compared to those of the NE and Vehl groups, there was a lower ABR threshold value in the Rd group after noise exposure (*p* < 0.01). With the extension of time, the hearing threshold of guinea pigs recovered gradually. There was no significant difference in the ABR threshold values between the NE and Vehl groups after noise exposure (*p* > 0.05), indicating that glycerin had no influence on NIHL treatment.

### DPOAE Amplitudes

The DPOAE amplitudes were essentially similar among the groups before noise stimulation. After intense noise exposure, the DPOAE amplitudes of the NE, Rd, and Vehl groups significantly reduced compared to those of the Con group (*p* < 0.01). Moreover, the amplitudes in the Rd group significantly increased after noise exposure compared to those in the NE and Vehl groups (*p* < 0.01). There was no significant difference in the DPOAE amplitudes between the NE and Vehl groups (*p* > 0.05). Frequency distribution analysis revealed that the DPOAE amplitudes in the NE, Rd, and Vehl groups were reduced in the high-frequency region of 4–8 kHz. The DPOAE amplitudes recorded 1 h after noise exposure are summarized in Table 3.

**TABLE 3:** DPOAE amplitudes of guinea pigs in each group after 1 h noise exposure (dB, *n* = 24).

Group	500	750	1k	2k
Con	8.44 ± 4.13^##^	7.94 ± 3.49^##^	10.79 ± 4.51^##^	10.94 ± 4.82^##^
NE	−3.22 ± 5.45**	−4.28 ± 5.18**	−3.94 ± 4.42**	−6.06 ± 4.13**
Rd	1.33 ± 3.36	1.94 ± 4.15	0.11 ± 4.27	−0.50 ± 3.88
Vehl	−3.17 ± 4.38**	−4.06 ± 4.96**	−3.56 ± 3.47**	−5.94 ± 3.67**

	**3k**	**4k**	**6k**	**8k**

Con	10.28 ± 4.03^##^	6.17 ± 3.19^##^	15.89 ± 4.25^##^	25.67 ± 4.45^##^
NE	−7.06 ± 2.65**	−12.94 ± 7.02**	−7.67 ± 6.81**	−6.18 ± 6.14**
Rd	−1.83 ± 4.94	−3.94 ± 4.63	−0.33 ± 4.67	4.28 ± 3.16
Vehl	−6.83 ± 2.98**	−11.06 ± 4.86**	−6.83 ± 5.61**	−6.89 ± 4.52**

The values are presented as the means ± SE. ^##^Con group vs. NE group: p < 0.01,**Rd group vs. NE group or Vehl group: p < 0.01.

### Tissue Section Staining

**FIGURE 2:**
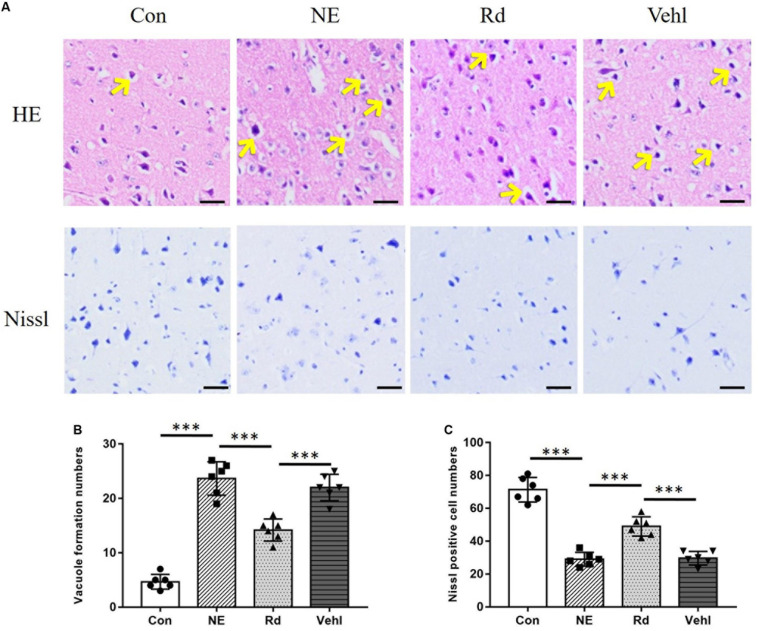
Original images **(A)** and summarized data **(B,C)** of HE or Nissl staining in the auditory cortex of all groups after exposure to 115 dB (A) noise. The arrows indicate the vacuole formation in the neuron cells; magnification ×400, scale bar = 50 μm. *N* = 6 in HE or Nissl staining. The values are presented as the means ± SE. ***p < 0.001.

Hematoxylin–eosin and Nissl staining revealed structural changes in the guinea pig auditory cortex neuron cells after noise exposure (Figure 2). Compared to the regular-shaped evenly stained normal cells of the Con group, HE staining showed morphological changes in the auditory cortex neuronal cells of the NE and Vehl groups, including swollen cells, vacuole formation, and nuclei deviation from the center or no nuclei. Nissl positive cells of the NE and Vehl groups were significantly decreased compared to those of the Con group (*p* < 0.01). Furthermore, noise-induced neuronal apoptosis and Nissl positive cells in the auditory cortex were significantly corrected by GSRd treatment (*p* < 0.01).

**FIGURE 3:**
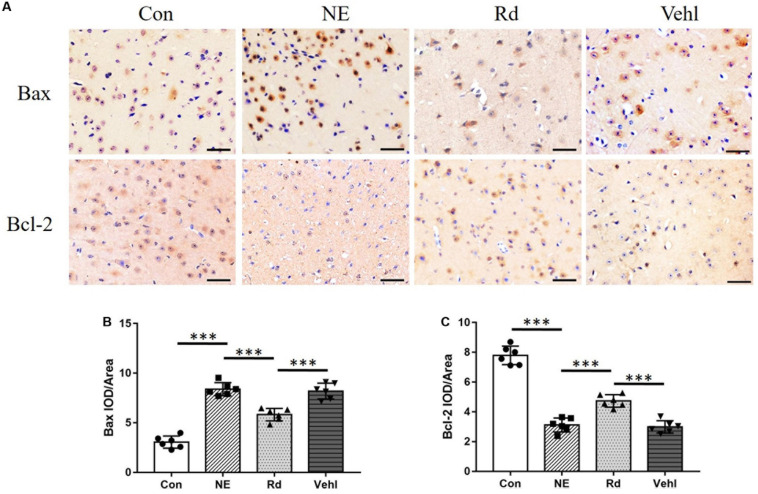
Original images **(A)** and summarized data **(B,C)** of immunohistochemical staining (IHC) of Bax and Bcl-2 in the auditory cortices of all groups after exposure to 115 dB (A) noise. Magnification ×400, scale bar = 50 μm. *N* = 6 in IHC staining. The values are presented as the means ± SE. ***p < 0.001.

The pro-apoptotic Bax and anti-apoptotic Bcl-2 are crucial mitochondrial proteins that regulate the intrinsic pathway of apoptosis. IHC analyses (Figure 3) revealed that the Bax integrated optical density (IOD) in the auditory cortices in the NE and Vehl groups was higher than that of those in the Con group (*p* < 0.01), and was subsequently significantly reduced by GSRd treatment (*p* < 0.01). Conversely, the IOD of Bcl-2 in the NE and Vehl groups was lower than that in the Con group (*p* < 0.01), which was subsequently increased by GSRd treatment (*p* < 0.01).

### RT-qPCR

**FIGURE 4:**
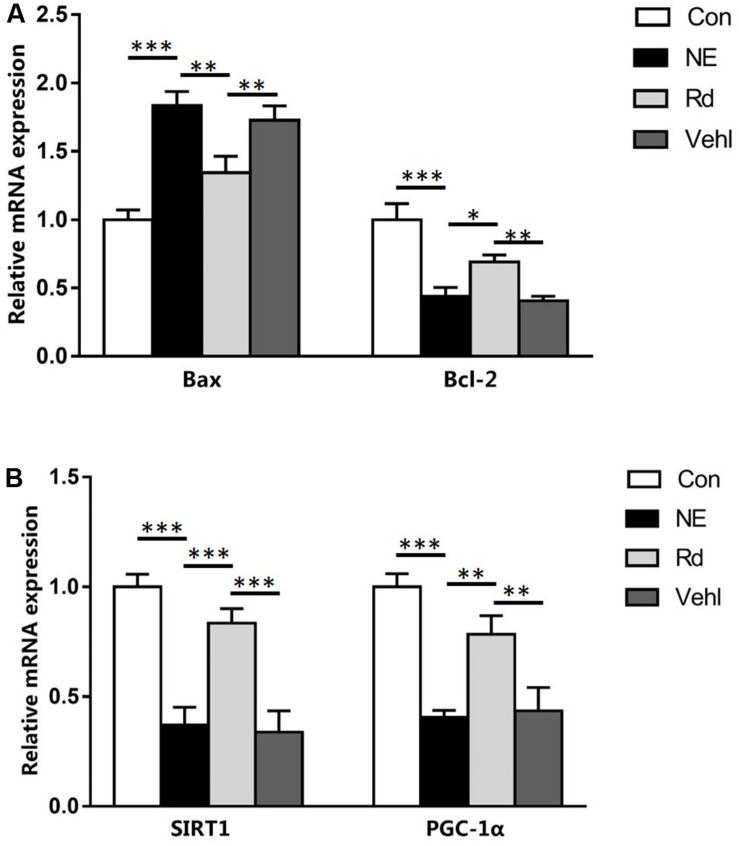
Summarized data showing the levels of Bax, Bcl-2 **(A)**, SIRT1, and PGC-1α **(B)** in the auditory cortices of guinea pigs in the four groups. *N* = 6 in each group. The values are presented as the means ± SE. *P < 0.05, **p < 0.01, ***p < 0.001.

Compared to the Con group, there was an increase in the Bax mRNA levels in the NE, Rd, and Vehl groups (*p* < 0.05); however, there was a reduction in the anti-apoptotic Bcl-2, SIRT1, and PGC-1α levels (*p* < 0.05). Moreover, there was a significant reduction of Bax mRNA level (*p* < 0.01), and elevation of Bcl-2, SIRT1, and PGC-1α mRNA levels (*p* < 0.05) in the Rd group compared to those in the NE and Vehl groups. Data are summarized in Figure 4.

### Western Blotting

**FIGURE 5:**
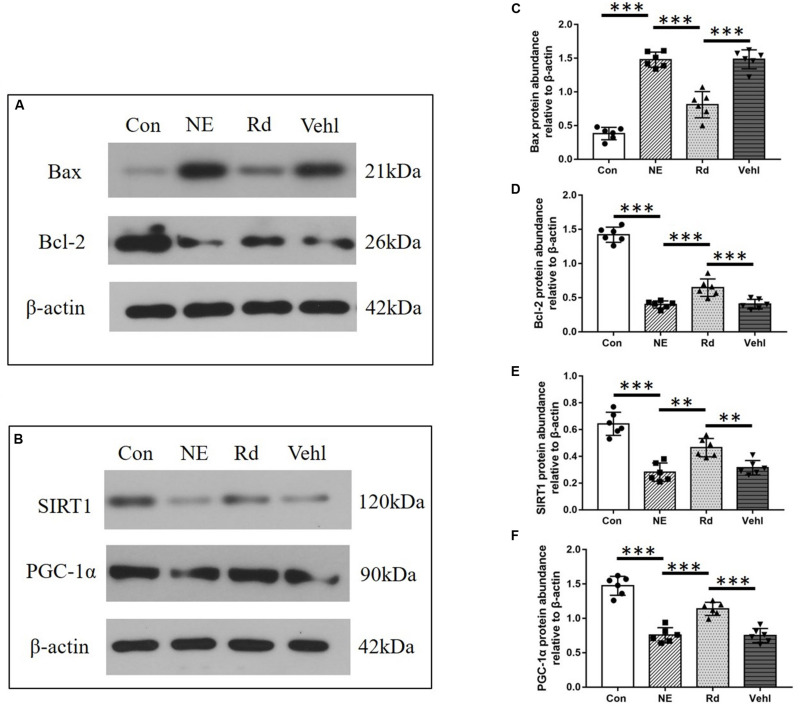
Original images **(A,B)** and summarized data **(C–F)** showing the levels of Bax, Bcl-2, SIRT1, and PGC-1α in the auditory cortices of guinea pigs in the four groups. *N* = 6 in each group. The values are presented as the means ± SE. **p < 0.01, ***p < 0.001.

A representative western blot is shown in Figure 5. Compared to the Con group, there was a significant increase in the Bax protein levels in the NE, Rd, and Vehl groups (*p* < 0.05); however, there was a significant reduction in the Bcl-2, SIRT1, and PGC-1α protein levels (*p* < 0.05). Moreover, GSRd up-regulated Bcl-2 expression and down-regulated Bax expression (*p* < 0.01). The Rd group also showed elevated SIRT1 and PGC-1α protein levels (*p* < 0.05) as compared to the NE and Vehl groups.

### SOD Activity, MDA, and GSH-Px Level

**FIGURE 6:**
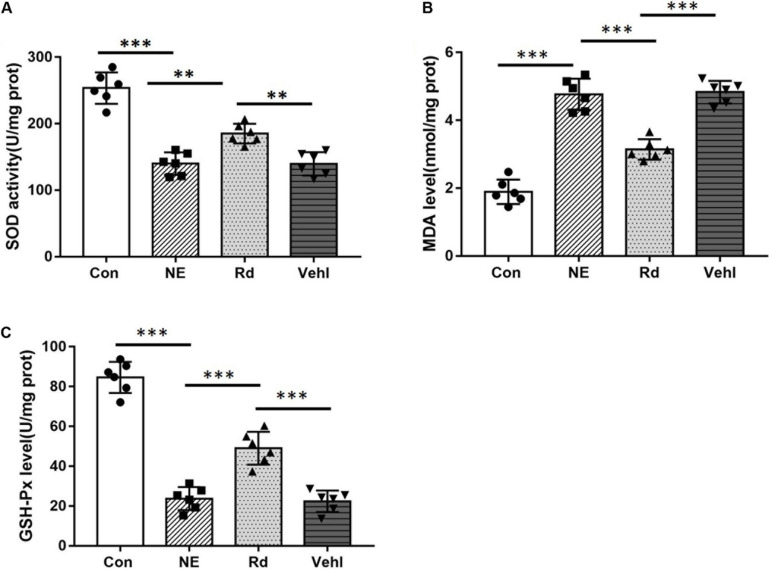
SOD activity **(A)** and MDA **(B)** and GSH-Px **(C)** levels in the four groups after noise exposure. **p < 0.01, ***p < 0.001.

Compared to the Con group, SOD levels in the NE, Rd, and Vehl groups were down-regulated by 47.3% (*p* < 0.01), 25.5% (*p* < 0.05), and 46.6% (*p* < 0.01), respectively. The corresponding GSH-Px levels were down-regulated by 75.0% (*p* < 0.01), 45.8% (*p* < 0.05), and 76.4% (*p* < 0.01), respectively. The corresponding MDA levels were up-regulated by 2.52 (*p* < 0.01), 1.63 (*p* < 0.05), and 2.50 times (*p* < 0.01), respectively. Moreover, immediately after noise exposure, we observed significantly increased SOD and GSH-Px levels (*p* < 0.05) and decreased MDA levels (*p* < 0.01) in the auditory cortices of guinea pigs in the Rd group compared with those in the NE and Vehl groups. Data are summarized Figure 6.

## Discussion

**FIGURE 7:**
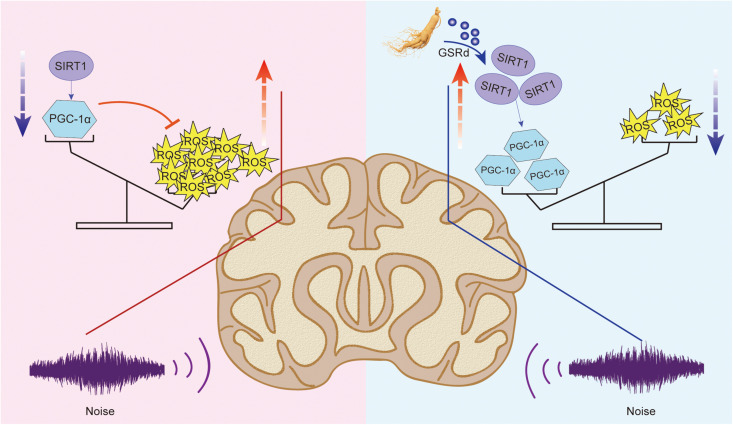
Protective mechanism of GSRd on auditory cortex injury induced by military helicopter noise.

The results of this study showed that GSRd significantly ameliorates auditory cortex injury associated with military aviation NIHL in guinea pigs by activating the SIRT1/PGC-1α signaling pathway (Figure 7).

The development of modern separation and analysis technology has allowed the elucidation of the ginseng chemical constituents. Ginsenoside is the main bioactive substance of ginseng and has been confirmed to possess various biological properties, such as anti-cancer, anti-fatigue, and stress-resistance properties. Until now, more than fifty ginsenoside monomers from Ginseng plants have been isolated and identified (Leung and Wong, 2010). Their basic structures are tetracyclic steroid nuclei with 17 arranged carbon atoms. According to their steroidal structure and the number of hydroxyl groups/sugar moieties attached to C-3 or C-6, ginsenoside monomers can be classified into three types: protopanaxadiol, protopanaxatriol, and oleanolic acid derivate. Protopanaxadiol and protopanaxatriol are dammarane-type saponins, which are the most important active ingredients in ginsenoside. GSRd is a protopanaxadiol among the dammarane-type saponins. Intestinal enzymes can metabolize high content GSRb1 to GSRd, which can then be absorbed and used by the body. Studies have shown that GSRd has strong biological activity and shows unique protective effects on the cardiovascular system (Zhang et al., 2019), immune system (Zhu et al., 2014), alimentary system (Liu et al., 2018; Phi et al., 2019), and central nervous system (Cong and Chen, 2016). Several animal studies have shown that GSRd can improve motor system function, reduce striatum injury area and cerebral infarction volume, have neurotrophic and protective effects on neurodegenerative diseases, such as Alzheimer’s disease and Parkinson’s disease, and improve neurological dysfunction caused by ischemic stroke (Ye et al., 2009; Wang et al., 2013). Other animal studies have shown that Rb1, the main monomer of GS, can prevent damage to spiral ganglion cells caused by vestibular dysfunction and cochlear ischemia (Fujita et al., 2007; Tian et al., 2013), and that Compound K monomer can alleviate noise-induced hearing impairment in Institute for Cancer Research (ICR) mice (Hong et al., 2011). Given that ginseng can improve neurodegenerative diseases and that the above-mentioned monomers have certain therapeutic effects on NIHL, we sought to determine whether GSRd also exerts neuroprotective effects on noise-induced auditory system damage.

Free radicals and oxidative stress play an important role in the pathogenesis of NIHL. Previous studies have confirmed that noise stimulation leads to the production of a wide range of free radicals in cochlear tissues and neurons, such as hydroxyl, hydrogen peroxide, superoxide anion, and NO-related free radicals (Rajguru, 2013; Tuerdi et al., 2017; Han et al., 2018). The free radicals produced by noise-induced oxidative stress can destroy proteins, lipids, and DNA when they exceed the endogenous antioxidant capacity, triggering cell death due to necrosis and apoptosis. This could subsequently destroy the auditory system structure and affect normal sensation and transmission functions, resulting in noise-induced sensorineural deafness, tinnitus, and auditory hypersensitivity (Henderson et al., 2006; Choi et al., 2014; Tuerdi et al., 2017). It has been reported that early use of free radical scavengers can reduce free radical production in the brain and alleviate noise-induced damage to the auditory system (Choi et al., 2014; Lu et al., 2014).

Initially, noise-induced damage occurs in the peripheral auditory system; however, under intense noise stimulation, metabolic and anatomical changes, including reduction of dendritic count, changes in neurotransmitter levels, and impairment of memory and cognition, also occur in the auditory cortex (Wankhar et al., 2017). Initially, bioinformatics analysis suggested that both GSRd treatment and SIRT1 activation exhibited alleviation of oxidative stress and anti-apoptosis effects; therefore, we hypothesized that SIRT1 signaling pathway activation is involved in the pharmacological effect of GSRd. Then, we observed neuronal apoptosis changes after noise exposure, including cell swelling, widespread vacuole formation, nuclei deviation from the center, and presence of several pyknotic nuclei. The reduced Nissl positive cells in the NE group indicated negated protein synthesis within the auditory cortex. Likewise, IHC analyses revealed reduced IOD of Bcl-2 and increased IOD of Bax in the NE group. Based on these findings, we expected to observe that GSRd had an effect on noise-induced structural and functional damage of the auditory cortex. Our results showed that after noise exposure, the ABR thresholds in the Rd group were significantly lower than those in the NE and Vehl groups, and that the decreases in the DPOAE amplitudes in the Rd group were lower than those in the NE and Vehl group. Morphologic changes and neuron apoptosis were also reversed after GSRd treatment, indicating that GSRd could improve NIHL to a certain extent.

Furthermore, we assessed the physiological influence of noise or GSRd treatment through SIRT1/PGC-1α signaling pathway activation. Sirtuin 1, which is short term for silent information regulator 1 (SIRT1), is a nicotinamide adenine dinucleotide-dependent deacetylase. It acts as a sensor to regulate intracellular oxidative stress and can activate antioxidant defense pathways to significantly reduce ROS levels (Seo et al., 2012; Xiong et al., 2014; Xue et al., 2016). SIRT1 also deacetylates many proteins involved in transcription activities, including p53 (Xiong et al., 2015), nuclear factor-kappa B (NF-κB) (Yeung et al., 2004), FOXO (Wang Y.Q. et al., 2015), and proliferator-activated receptor-gamma coactivator 1α (PGC-1α). PGC-1α is a key enzyme involved in mitochondrial biogenesis and oxidative metabolism (Lagouge et al., 2006). Recent studies have shown that activation of the SIRT1/PGC-1α signaling pathway can promote the expression of antioxidant enzymes and improve free radicals associated disorders (Wang S.J. et al., 2015; Bu et al., 2017) such as Alzheimer disease and type 2 diabetes (Satoh et al., 2013; Herskovits and Guarente, 2014). NIHL involves excessive free radicals, with studies showing the involvement of SIRT1 in its pathogenesis (Xiong et al., 2017).

Our results showed that there were significantly lower levels of SIRT1 and PGC-1α mRNA and proteins in the auditory cortices of guinea pigs in the NE and Rd groups compared with those in the Con group, suggesting that noise-induced auditory cortex damage may involve down-regulation of the SIRT1/PGC-1α signaling pathway, which is reversed by GSRd treatment through enhancement of auditory cortex SIRT1 activity and reduction of oxidative stress. Further, the mRNA and protein levels of Bax were increased, while those of Bcl-2 were reduced after noise exposure, which indicated that the intrinsic pathway of apoptosis was promoted by noise exposure. More importantly, GSRd treatment suppressed noise-induced neuronal apoptosis in the auditory cortex.

Superoxide dismutase is an important enzyme that directly scavenges oxygen free radicals. When its activity decreases, the *in vivo* concentration of free radicals increases. Therefore, measuring SOD activity in tissues may be used to indicate the concentration of oxygen free radicals. MDA is a stable product of lipid peroxidation induced by oxygen free radicals, and indirectly reflects the severity of the free radical attack on human cells (Su et al., 2017). GSH-Px is an important peroxidase that is widely distributed throughout the body and catalyzes the reduction of reduced glutathione to hydroperoxide; therefore, it can remove harmful peroxide metabolites, block the lipid peroxidation chain reaction, and protect the cell membrane structure and function. We observed a significant increase in the SOD and GSH-Px levels and a decrease in MDA levels in the auditory cortices in the Rd group, compared to those observed in the NE and Vehl group. We speculate that noise-induced auditory cortex damage may involve a decrease in the brain tissue’s ability to scavenge free radicals, which results in an increase in the concentration of ROS and lipid peroxidation. It is worth noting that GSRd can partly improve the ability to scavenge free radicals.

Several limitations of this study should be acknowledged. First, although this animal study has shown that ginsenoside is effective, there is still a need for multi-stage clinical studies to confirm this in humans. This is because of the many restrictions against the direct translation of animal studies to human. Second, we could not specify the specific mechanism of action. Third, the mode of administration and dose tolerance in humans are not clear (Kang and Chung, 2010). Finally, further studies are required to determine whether its combination with other ginsenoside monomers or other antioxidants could improve its efficacy in NIHL treatment.

## Conclusion

Our results demonstrate that GSRd ameliorates auditory cortex injury associated with military aviation NIHL in guinea pigs via activation of the SIRT1/PGC-1α signaling pathway. Therefore, GSRd can be a promising candidate therapy for NIHL.

## Data Availability Statement

All datasets generated for this study are included in the article/Supplementary Material.

## Ethics Statement

The animal study was reviewed and approved by the Institutional Animal Care and Use Committee of Air Force Medical University, China.

## Author Contributions

XC, YL, YC, and XW conceived and designed the methods. XC and SJ drafted the manuscript. XC, XX, JX, and SD performed ABR and DPOAE tests. CL examined SOD, MDA, and GSH-Px levels. XC and YL performed HE and Nissl staining, immunohistochemistry analysis, RT-qPCR, and western blotting analysis. ZG conducted the statistics and interpreted results. XC, SJ, and HW revised the manuscript. XW took responsibility for the integrity of the data and the accuracy of data analysis. All authors contributed to the article and approved the submitted version.

## Conflict of Interest

The authors declare that the research was conducted in the absence of any commercial or financial relationships that could be construed as a potential conflict of interest.

## Footnotes

1 http://stitch.embl.de/

2 https://metascape.org/

## Supplementary Material

FIGURE S1 Percentage figure: GO enrichment analysis of GSRd from STITCH database.

FIGURE S2 GO enrichment analysis of GSRd from STITCH database.

FIGURE S3 KEGG enrichment analysis of GSRd from STITCH database.

FIGURE S4 Volcano plot of differentially expressed genes after SIRT1 activation.

TABLE S1 Gene ontology and pathway enrichment analysis of the up-regulated and down-regulated genes.

## Abbreviations

ABR: auditory brainstem response
DAB: 3,3′-diaminobenzidine
DPOAE: distortion product otoacoustic emission
GSH-Px: glutathione peroxidase
GSRd: ginsenoside Rd
HE: hematoxylin–eosin
HRP: horseradish peroxidase
IOD: integral optical density
MDA: malondialdehyde
NIHL: noise-induced hearing loss
ROS: reactive oxygen species
SOD: superoxide dismutase
SPL: sound pressure level.
