# Supplementary figures and images for: Ginsenoside Rd Ameliorates Auditory Cortex Injury Associated With Military Aviation Noise-Induced Hearing Loss by Activating SIRT1/PGC-1α Signaling Pathway
Source: Front Physiol. 2020 Jul 21;11:788. doi: 10.3389/fphys.2020.00788 (PMC7385399; doi:10.3389/fphys.2020.00788)

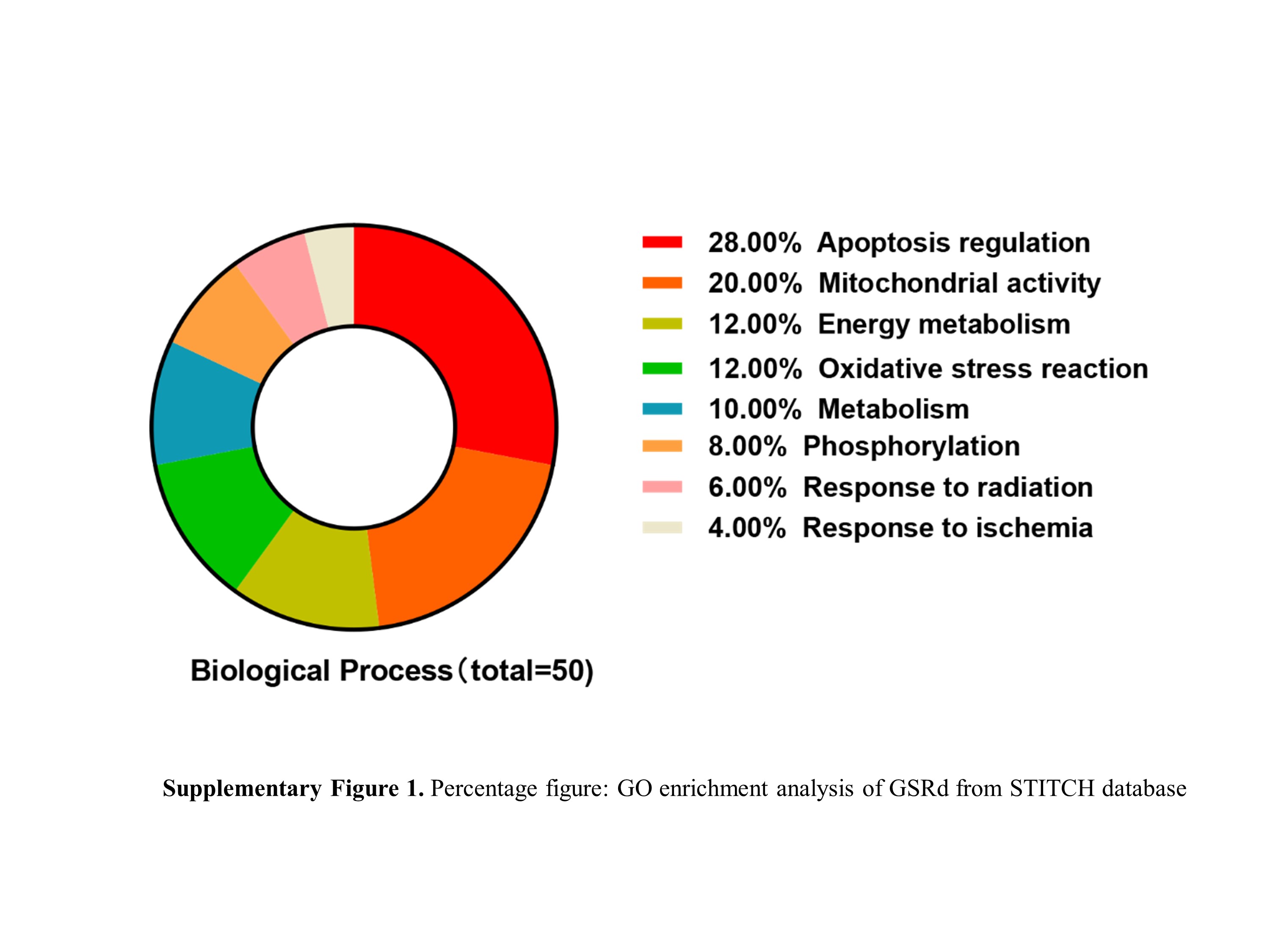

Supplement: FIGURE S1 — Percentage figure: GO enrichment analysis of GSRd from STITCH database. [file Image_1.JPEG]

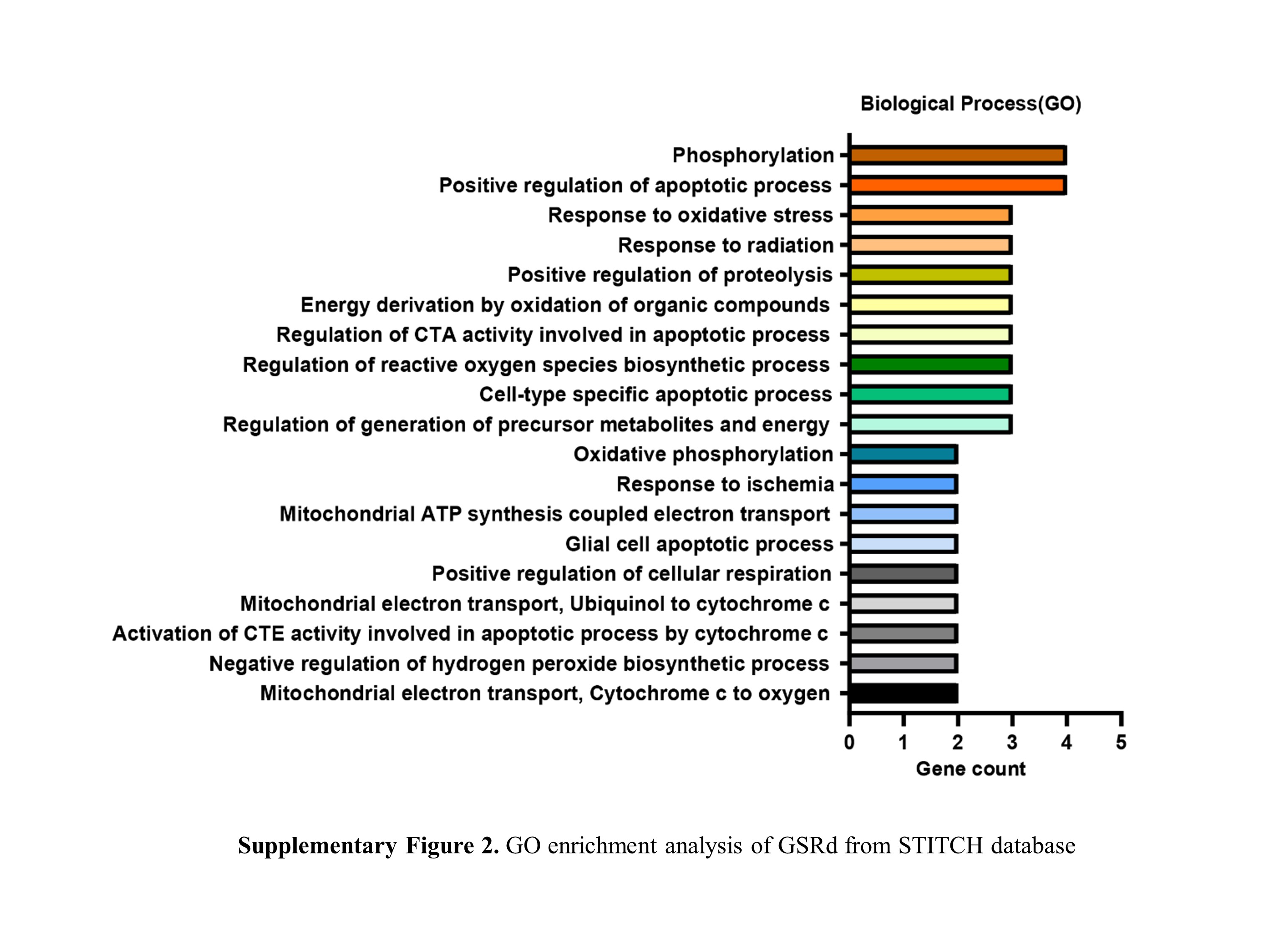

Supplement: FIGURE S2 — GO enrichment analysis of GSRd from STITCH database. [file Image_2.JPEG]

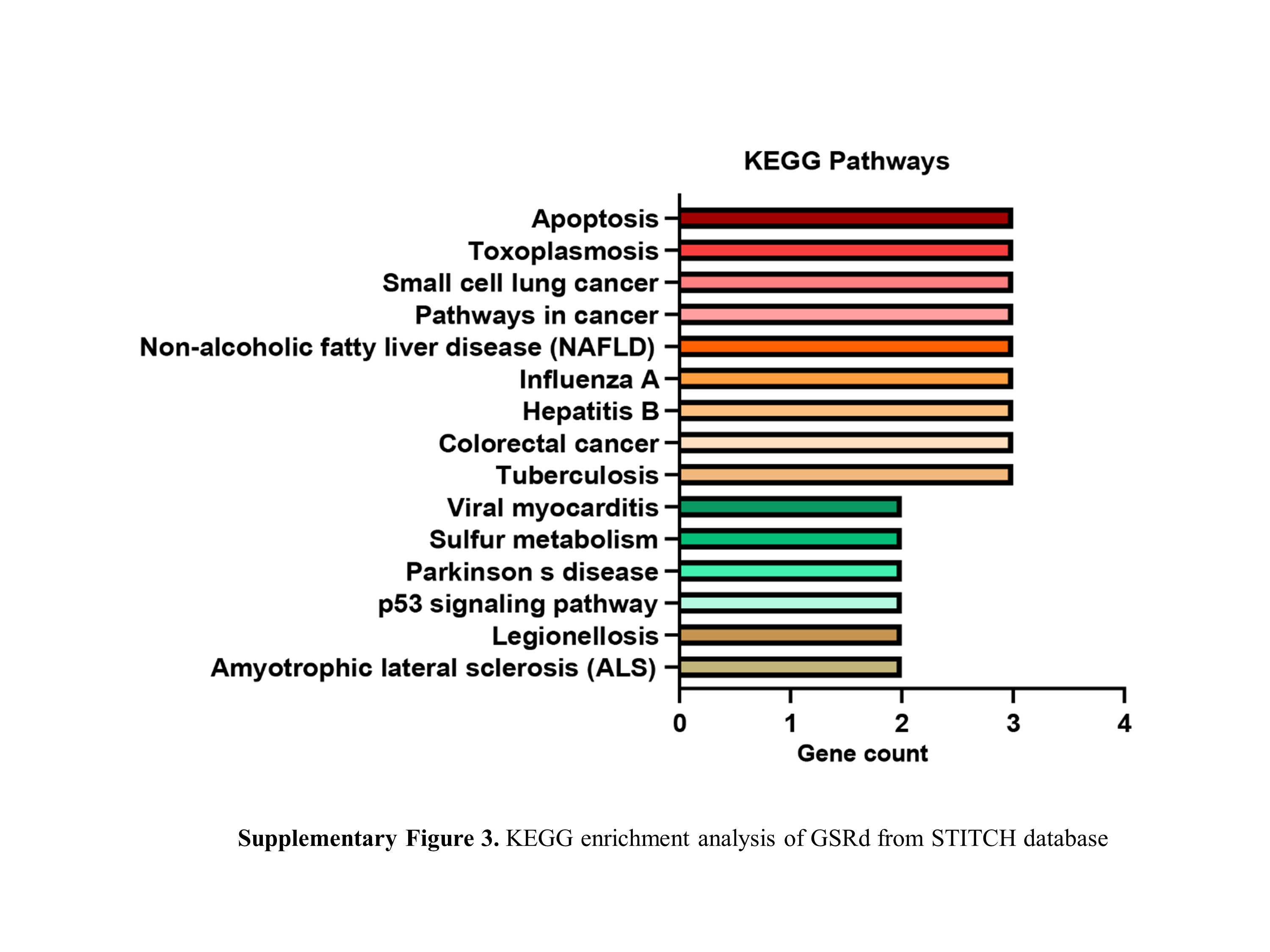

Supplement: FIGURE S3 — KEGG enrichment analysis of GSRd from STITCH database. [file Image_3.JPEG]

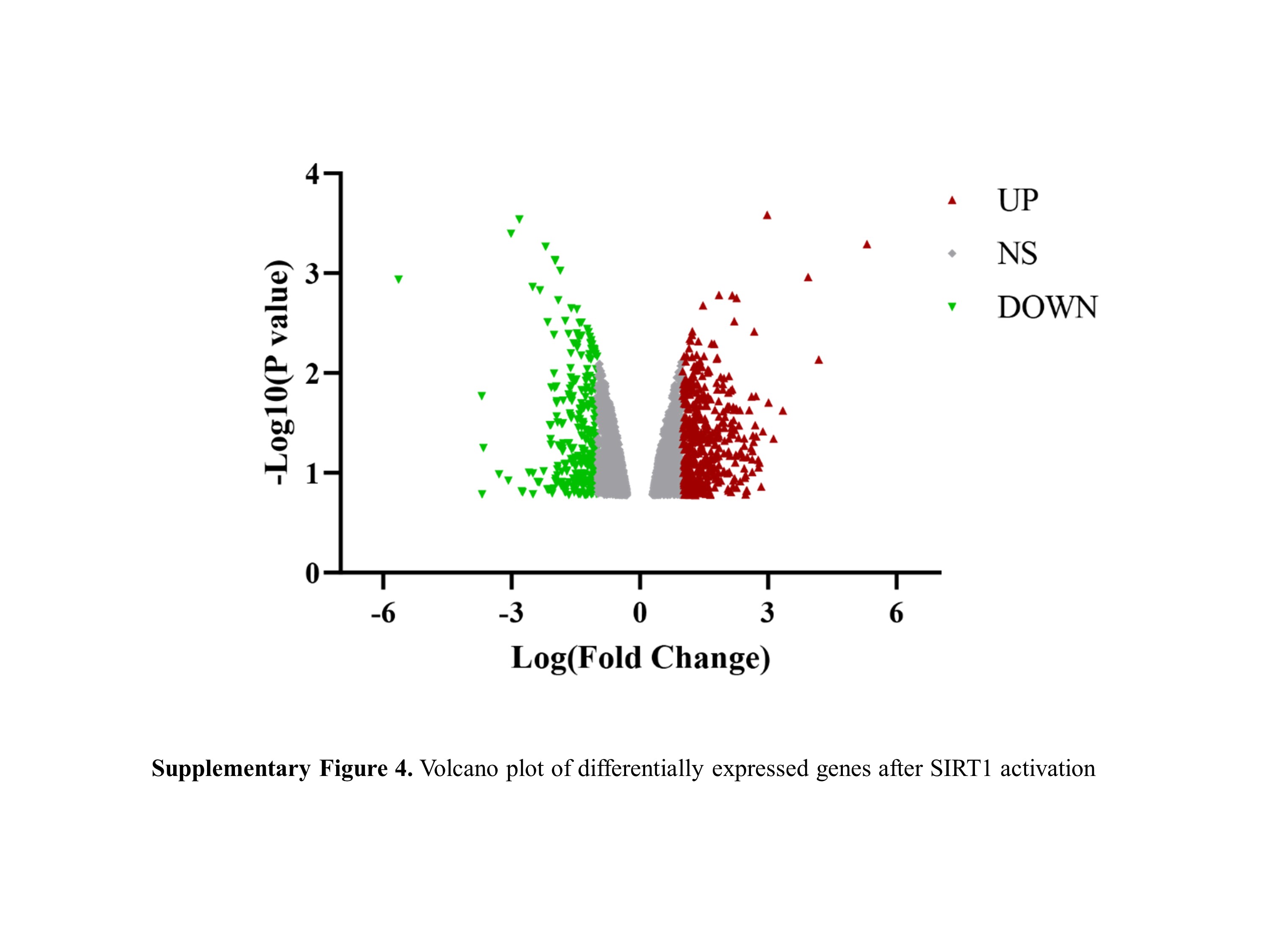

Supplement: FIGURE S4 — Volcano plot of differentially expressed genes after SIRT1 activation. [file Image_4.JPEG]

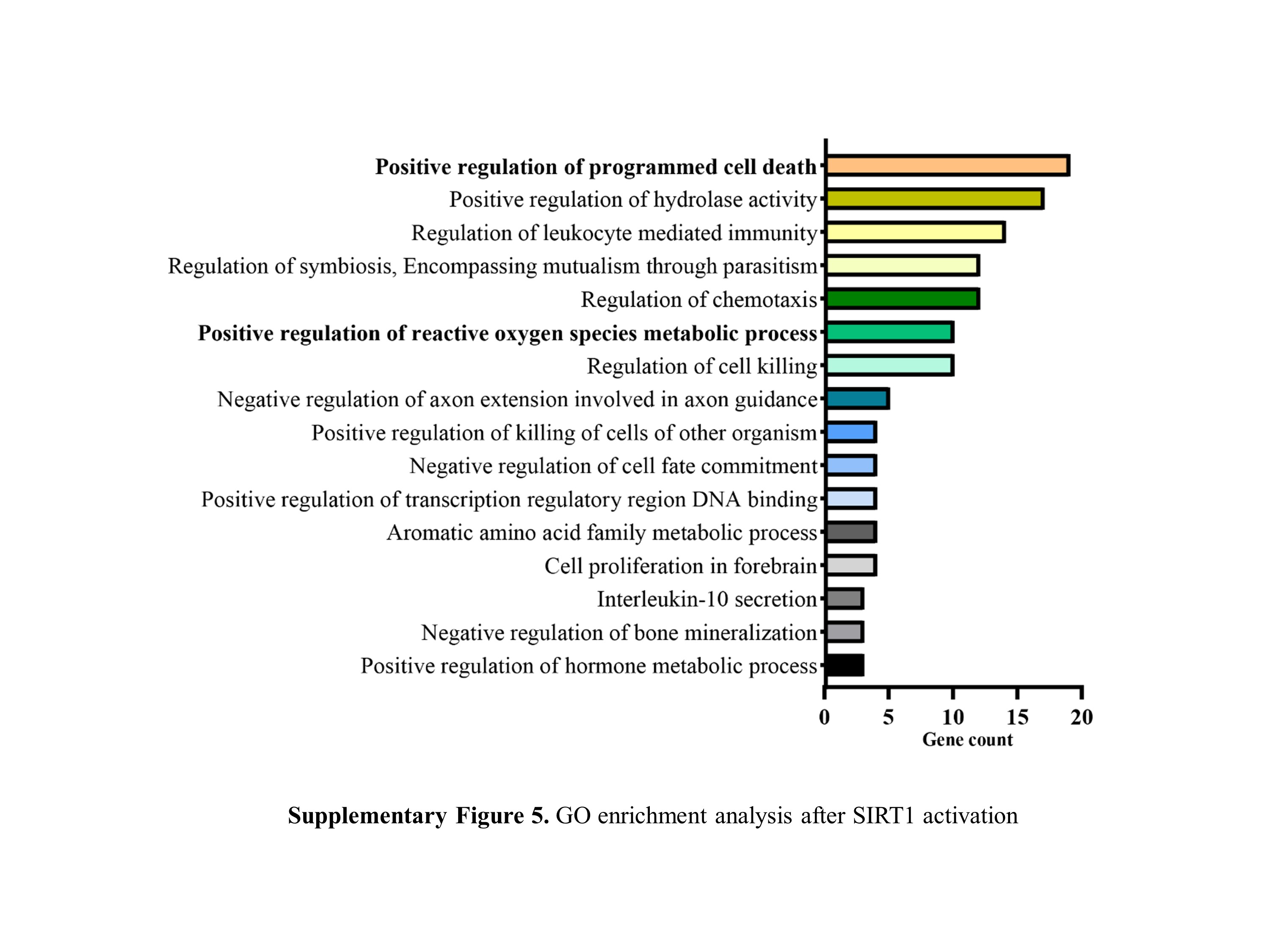

Supplement: Supplementary file 5 [file Image_5.JPEG]
